# Stage of colorectal cancer diagnosis for immigrants: a population-based retrospective cohort study in Ontario, Canada

**DOI:** 10.1007/s10552-021-01491-5

**Published:** 2021-08-31

**Authors:** A. K. Lofters, E. Gatov, H. Lu, N. N. Baxter, A. M. Corrado, S. J. T. Guilcher, A. Kopp, M. Vahabi, G. D. Datta

**Affiliations:** 1 Department of Family and Community Medicine, University of Toronto, Toronto, Canada; 2 Women’s College Hospital Research Institute, Toronto, Canada; 3 Peter Gilgan Centre for Women’s Cancers, Women’s College Hospital, 76 Grenville St., Toronto, ON M5S 1B2 Canada; 4 ICES, Toronto, Canada; 5 MAP Centre for Urban Health Solutions, St. Michael’s Hospital, Toronto, Canada; 6 Melbourne School of Population and Global Health, University of Melbourne, Melbourne, Australia; 7 Leslie Dan Faculty of Pharmacy, University of Toronto, Toronto, Canada; 8 Daphne Cockwell School of Nursing, Ryerson University, Toronto, Canada; 9 Department of Social and Preventive Medicine, Université de Montréal, Montreal, Canada; 10 Research Center of the University of Montreal Hospital Center (CR-CHUM), Montreal, Canada

**Keywords:** Immigrant health, Cancer stage, Colorectal cancer, Health disparities

## Abstract

**Background:**

Colorectal cancer (CRC) is the second most common cause of cancer death in Canada. Immigrants in Ontario, Canada’s most populous province, are known to have lower rates of CRC screening, but differences in stage of CRC diagnosis are not known.

**Methods:**

We utilized linked administrative databases to compare early (stage I–II) versus late (stage III–IV) stage of CRC diagnosis for immigrants versus long-term residents among patients diagnosed in Ontario between 2012 and 2017 (*n* = 37,717) and examined the association of immigration-related, sociodemographic, and healthcare-related factors with stage.

**Results:**

Almost 45% of those with CRC were diagnosed at a late stage. Immigrants were slightly more likely to be diagnosed at a late stage than their long-term resident counterparts [Adjusted relative risks (ARRs) 1.06 (95% CI 1.02–1.10)], but after adjusting for age and sex, this difference was no longer significant. In fully adjusted models, we observed a higher likelihood of late-stage diagnosis for people with the fewest co-morbidities (ARR 0.86 [95% CI 0.83–0.89]) and those with no visits to primary care (versus a high level of continuity of care) [ARR 1.07 (95% CI 1.03–1.12)].

**Conclusion:**

Immigrants were not more likely to have a late-stage CRC diagnosis after adjusting for relevant factors, but access to primary care and healthcare contact was significantly associated with diagnostic stage.

**Impact:**

Attachment to a primary care provider who provides regular preventive care may play a role in more favorable stage at diagnosis for CRC and thus should be a healthcare system priority.

## Introduction

Colorectal cancer (CRC) is the second most common cancer and the second most common cause of cancer death in Canada, with approximately 10,000 deaths per year [1]. Stage of diagnosis is strongly associated with survival, with stage IV survival estimated at only 11% [2]. In contrast, when diagnosed at stage I, survival is over 90% [2]. Unfortunately, 49% of all cases of CRC diagnosed in Canada between 2011 and 2015 were diagnosed at stage III or IV [2]. Evidence-based and targeted approaches are needed to reduce these avoidable deaths through screening, early identification, and treatment.

Screening for colorectal cancer can take several forms. In 2008, the Canadian province of Ontario implemented an organized CRC screening program, where the biennial use of fecal testing was recommended for average-risk adults aged 50–74 years [3]. Until 2019, Fecal occult blood test (FOBT) was recommended; however, Fecal immunochemical test (FIT) is now the recommended test. While the organized provincial program uses fecal-based testing, colonoscopy and flexible sigmoidoscopy are also used for screening in some settings and the program considers those who have had either test in the past ten years to be up to date on screening. Regardless of modality used, rates of CRC screening in the province are currently suboptimal, with an estimated 39% of eligible Ontarians overdue for screening [4]. This suboptimal uptake is of particular relevance considering that it has been estimated that CRC deaths can be reduced by 13% with regular fecal screening [5].

The reasons for suboptimal CRC screening uptake are not clear but research has shown lower uptake of CRC screening among immigrants versus Canadian-born residents in Ontario [6–8]. This inequality is particularly compelling as immigrants make up 28% of the Ontario population [9]. Importantly, CRC screening rates are not uniform across the province’s ethnoculturally diverse immigrant groups, with only 31% of East Asian immigrants being overdue for CRC screening versus 44% of immigrants from Eastern Europe and from South Asia [10]. Similarly, research has found that, although immigrants overall have a lower risk of developing CRC than those who are Canadian-born, immigrants from Eastern European countries have previously been shown to have a higher risk [11]. It is possible that inequalities in CRC screening according to immigrant status may lead to inequalities in stage of diagnosis of CRC. However, we cannot assume that patterns for stage of diagnosis will directly follow patterns of screening [12, 13]. While screening certainly plays a key role in stage of diagnosis for CRC, quality of care, structural factors (e.g., systemic discrimination), and biological factors due to shared geographic ancestry may also play a role and thus the association of stage of CRC diagnosis with immigrant status cannot be presumed to be known.

In this population-based retrospective cohort study, we utilized provincial-level administrative databases to compare early (stage I–II) versus late (stage III–IV) stage of diagnosis of CRC for immigrants versus long-term residents of Ontario among patients diagnosed with CRC between 2012 and 2017 and to examine the role of immigration-related, sociodemographic, and healthcare-related factors on diagnostic stage.

## Methods

### Study setting and data sources

Ontario is Canada’s most populous and multi-ethnic province with over 13 million residents, 28% of whom were foreign-born [9]. Ontario has a universal healthcare system which provides all permanent residents (and temporary residents with a work permit who have been employed full time for at least six months) with a health card and unique health card number that gives them free access to medically necessary healthcare including primary care and preventive services such as cancer screening. The province does not have a formal healthcare integration support system for newcomers. Ontario is also home to ICES, an independent, non-profit research institute whose legal status under Ontario’s health information privacy law allows it to collect and analyze healthcare and demographic data, without consent, for health system evaluation and improvement.

To construct the cohort, we utilized the Ontario Cancer Registry, which contains information on approximately 95% of all provincial cancer diagnoses [14]. To determine immigration status, we used the Immigration, Refugees and Citizenship Canada - Permanent Resident (IRCC-PR) dataset, which identifies Ontario immigrants who arrived in Canada from 1985 onward [15]. To ascertain patient demographics, we used the Registered Person’s Database, identifying age, sex, and residential postal code via health card information. To examine primary care, we used the Ontario Health Insurance Plan for physician billings and the Client Agency Program Enrollment for patient–provider rostering. To determine provider characteristics, we used the Corporate Provider Database and the ICES Physicians’ Database (IPDB), which record demographic information about Ontario’s physicians who are in active practice. Lastly, to capture health service utilization information, we used the National Ambulatory Care Reporting System for emergency department visits and ambulatory care and the Canadian Institute for Health Information Discharge Abstract Database for hospitalizations. These datasets were linked using unique encoded identifiers and analyzed at ICES.

### Study cohort

**Fig. 1:**
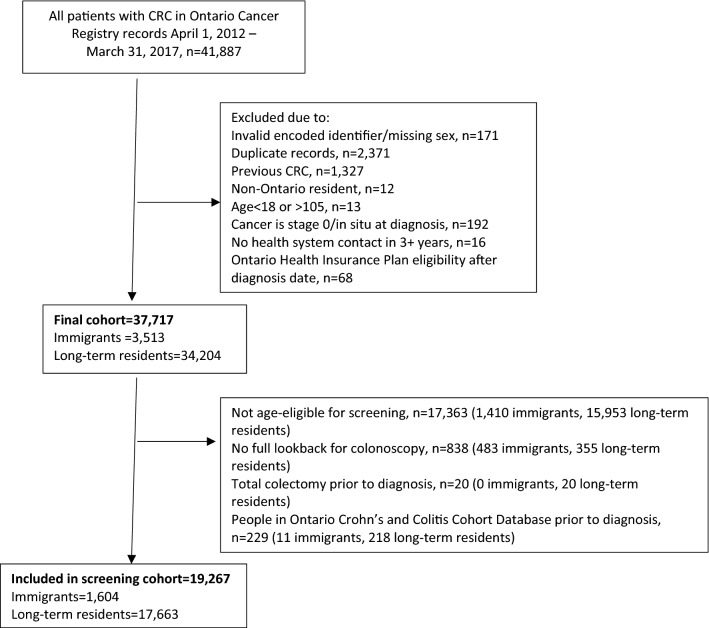
Creation of final cohort of 37,717 patients with colorectal cancer (CRC) and subset of 19,267 patients eligible for CRC screening

Our cohort consisted of adults 18 years and over who were diagnosed with incident CRC between 1 April 2012 and 31 March 2017 (Fig. 1). We excluded those who had invalid identifiers, duplicate records, a previous CRC diagnosis, those who were less than 18 years of age or over 105 years of age, were not residents of Ontario, did not have healthcare coverage for at least three years prior to diagnosis (and thus had limited available data), and whose cancer was stage 0/in situ. We then categorized the populations into “immigrants” and “long term residents” based on their inclusion in the IRCC-PR database. We categorized those not in the database as long-term residents, as this group would include both non-immigrants and immigrants who arrived prior to 1985. Thus, immigrants would have lived in Ontario for anywhere from 3 to 32 years by the study end date. Long-term residents would be expected to have lived in Ontario for a minimum of 32 years to as long as their entire lives. We also identified a subset of the cohort who were screen eligible at the time of diagnosis, i.e., 50–74 years of age at diagnosis without total colectomy prior to diagnosis and not in the Ontario Crohn’s and Colitis Cohort Database. The latter exclusion was made in order to limit the sample to those who are considered average risk for CRC and thus should follow population-level screening recommendations.

### Outcome

We used Ontario Cancer Registry data to determine the best available stage of diagnosis. The Collaborative Staging System is a unified data collection system used in Canada and the USA that is based on a set of pathological and clinical data items including tumor size, extension, lymph node status, and metastasis status [16]. Capture of Collaborative Stage for CRC became available at ICES in 2010. We used an algorithm whereby Collaborative Stage is determined where available as the stage of record for each case. If Collaborative Stage was not available, stage was based on physician staging from the regional cancer center. If neither was captured, stage was coded as missing. Where stage was missing, this was the result of limited stage work-up, limited documentation in the person’s health record, or both [17]. As of 2013, 90% of lung, female breast, colorectal, cervix, and prostate cancers in the Ontario Cancer Registry have complete staging information [17]. We further dichotomized stage into I–II (“early stage”) vs. III–IV (“late stage”), as is common in the literature [18–20] in order to group CRCs at differing probability of survival.

### Study variables

To describe the cohort, we used the Registered Persons’ Database to retrieve patient sex, age, and postal code at the time of diagnosis. Neighborhood-area income quintile was identified by linking the residential postal code to 2016 Census data on mean household income. To categorize co-morbidity, we use Aggregated Diagnosis Groups (ADGs) from the Johns Hopkins ACG® case-mix system (V10.0) [21], which identify morbidities from diagnosis codes in outpatient billing and inpatient hospital records. The ADGs represent groups of conditions with similar healthcare experience relating to attributes such as severity and duration of disease. Finally, using IRCC-PR data, we subcategorized immigrants by regions of origin, immigrant class, and by length of time in Canada at date of diagnosis. For region of origin, we used country of birth and classified those countries into regions based on a modified and previously published World Bank classification [10, 13, 22–24].

Using Ontario Health Insurance Plan physician billing data in the 6 to 30 months before diagnosis (a two-year period), we determined the number of primary care visits for each patient and examined their continuity of care based on the Usual Provider of Care index, which measures the proportion of all primary care visits that were made to the provider most frequently visited among those with at least three primary care visits in the two-year period [25]. We excluded primary care visits during the 0 to 6 months before diagnosis because they may reflect a peri-diagnostic interval as opposed to usual care [26]. Continuity of care was defined as Usual Provider of Care index of 75% or greater. We then used the IPDB to categorize primary care physician sex and region of training, as these physician-level variables have previously been associated with CRC screening in Ontario [24].

To examine screening status for screen-eligible members of the cohort, we used a lookback period starting six months prior to the date of diagnosis so as to not inadvertently include fecal tests or colonoscopies that were performed for case-finding rather than screening purposes. Screening status for CRC was determined from physician billing data and was defined as “up-to-date” if the individual received fecal testing in the previous 2 years from lookback or sigmoidoscopy/colonoscopy in the previous 10 years from lookback, “ever screened but overdue” if the individual had fecal testing, sigmoidoscopy, or colonoscopy in the past according to available data but was not up to date, or “no record of screening”. Individuals without complete data for 10 years prior to lookback were not included in this subgroup of screen-eligible members of the cohort.

### Analysis

To describe the cohort at the time of diagnosis, we compared sociodemographic and clinical characteristics, as well as CRC screening history and primary care provider characteristics between immigrants and non-immigrants using chi-square tests and standardized differences, which are independent of sample size (a standardized difference of > 0.10 is considered clinically important) [27]. We further stratified each group (immigrants and non-immigrants) by cancer stage, since we hypothesized a priori that the relationship between patient characteristics and cancer stage may differ among immigrants and non-immigrants.

To examine the risk of diagnosis at a late stage among immigrants compared to non-immigrants, we implemented modified Poisson regressions with robust standard errors (which yield relative risk estimates) first unadjusted, then age and sex-adjusted (age as a continuous variable), and then fully adjusted for age, sex, neighborhood-area income quintile, number of ADG co-morbidities, and primary care characteristics, including the number of prior primary care visits, continuity of primary care, and primary care provider sex and region of training, dichotomized as Canadian versus non-Canadian.

Statistical analyses were conducted using SAS software (version 9.4). The study was approved by the Research Ethics Board at Unity Health Toronto.

## Results

Of 41,887 patients identified with CRC between 2012 and 2017, there were 37,717 people included in the final cohort, 3,513 (9.3%) of whom were identified as immigrants (Fig. 1). Descriptive characteristics of the study population are shown in Table 1. Males made up 54.5% of the total cohort. Age at diagnosis was significantly different between immigrants and long-term residents, with immigrants diagnosed at 62.6 years on average versus 70.1 years for long-term residents [standardized difference (SD) = 0.55]. Immigrants were more likely to live in the lowest income neighborhoods (27.6% vs. 20.1%, SD = 0.18), had fewer co-morbidities at diagnosis, and had less continuity of care with their primary care physicians. They also were more likely than long-term residents to see a female primary care physician (33.3% vs. 28.0%, SD = 0.12) or one trained outside of Canada (47.9% vs. 24.9%, SD = 0.49). Immigrants in the cohort had been in Canada a median of 19 years at the time of diagnosis and more than 60% were from either East Asia and the Pacific or Europe and Central Asia. Of the 19,267 cohort members who were eligible for screening and for whom we had at least 10 years of available data prior to diagnosis, a significantly higher proportion of immigrants than long-term residents (41.9% vs. 36.2%) had no record of CRC screening at any time prior to the six months before diagnosis. This difference was largely driven by lower use of colonoscopy among immigrants. Less than one-third of patients in the overall cohort were up to date on any type of CRC screening at six months prior to diagnosis, 36.7% had no record of screening, and 30.7% had been screened in the past but were overdue. Among this latter group, the median time to last colonoscopy was 4753 days (13.0 years) and the median time to last FOBT was 1474 days (4.0 years).

**Table 1:** Descriptive characteristics by immigrant status of 37,717 people in Ontario diagnosed with colorectal cancer between 2012 and 2017

Characteristics	Immigrants (*n* = 3,513)	Long-term residents (*n* = 34,204)	Standardized difference	*p*-value	Total (*n* = 37,717)
Sex					
Female	1,602 (45.6%)	15,544 (45.4%)	0.00	0.859	17,146 (45.5%)
Male	1,911 (54.4%)	18,660 (54.6%)	0.00		20,571 (54.5%)
Age at diagnosis					
Mean ± SD	62.57 ± 14.14	70.10 ± 13.07	0.55	< .001	69.39 ± 13.35
Median (IQR)	62 (52–73)	71 (61–80)	0.55	< .001	70 (60–80)
Age group					
< 50	614 (17.5%)	2,212 (6.5%)	0.34	< .001	2,826 (7.5%)
50–74	2,103 (59.9%)	18,251 (53.4%)	0.13		20,354 (54.0%)
75–84	590 (16.8%)	9,043 (26.4%)	0.24		9,633 (25.5%)
85+	206 (5.9%)	4,698 (13.7%)	0.27		4,904 (13.0%)
Neighborhood income quintile					
Missing	7 (0.2%)	71 (0.2%)	0.00	< .001	78 (0.2%)
Quintile 1 (lowest)	971 (27.6%)	6,860 (20.1%)	0.18		7,831 (20.8%)
Q2	752 (21.4%)	7,176 (21.0%)	0.01		7,928 (21.0%)
Q3	701 (20.0%)	6,874 (20.1%)	0.00		7,575 (20.1%)
Q4	627 (17.8%)	6,615 (19.3%)	0.04		7,242 (19.2%)
Q5 (highest)	455 (13.0%)	6,608 (19.3%)	0.17		7,063 (18.7%)
Number of John’s Hopkins ADG co-morbidities					
Mean ± SD	6.50 ± 3.29	7.07 ± 3.65	0.16	< .001	7.02 ± 3.62
Median (IQR)	6 (4–9)	7 (4–9)	0.15	< .001	7 (4–9)
0–5	1,444 (41.1%)	12,551 (36.7%)	0.09	< .001	13,995 (37.1%)
6–9	1,433 (40.8%)	13,219 (38.6%)	0.04		14,652 (38.8%)
10+	636 (18.1%)	8,434 (24.7%)	0.16		9,070 (24.0%)
No. PCP visits 6–30 months < index—all primary care providers					
Mean ± SD	8.02 ± 7.83	7.43 ± 7.56	0.08	< .001	7.49 ± 7.59
Median (IQR)	6 (3–11)	6 (2–10)	0.09	< .001	6 (2–10)
No. PCP visits 6–30 months < index—patient’s usual provider of care					
Mean ± SD	5.76 ± 6.49	5.66 ± 6.49	0.02	0.388	5.67 ± 6.49
Median (IQR)	4 (1–8)	4 (1–8)	0.02	0.369	4 (1–8)
Usual provider of care (UPC) index					
Missing	85 (2.4%)	1,206 (3.5%)	0.07	< .001	1,291 (3.4%)
0 visits	252 (7.2%)	2,428 (7.1%)	0.00		2,680 (7.1%)
1–2 visits	599 (17.1%)	6,477 (18.9%)	0.05		7,076 (18.8%)
UPC≤75%	728 (20.7%)	5,523 (16.1%)	0.12		6,251 (16.6%)
UPC > 75%	1,849 (52.6%)	18,570 (54.3%)	0.03		20,419 (54.1%)
Primary care provider sex					
Female	1,169 (33.3%)	9,563 (28.0%)	0.12	< .001	10,732 (28.5%)
Male	2,247 (64.0%)	23,314 (68.2%)	0.09		25,561 (67.8%)
Missing	97 (2.8%)	1,327 (3.9%)	0.06		1,424 (3.8%)
Primary care provider country of training					
Canada	1,664 (47.4%)	23,766 (69.5%)	0.46	< .001	25,430 (67.4%)
Not Canada	1,683 (47.9%)	8,533 (24.9%)	0.49		10,216 (27.1%)
Missing	166 (4.7%)	1,905 (5.6%)	0.04		2,071 (5.5%)
Immigrant category					
Economic class immigrants	1,208 (34.4%)	–	–	–	–
Sponsored family immigrants	1,654 (47.1%)	–	–	–	–
Other immigrants	84 (2.4%)	–	–	–	–
Resettled refugee and protected person in Canada	567 (16.1%)	–	–	–	–
World bank region of origin					
East Asia and Pacific	1,102 (31.4%)	–	–	–	–
Europe and Central Asia	1,027 (29.2%)	–	–	–	–
Latin America and the Caribbean	410 (11.7%)	–	–	–	–
Middle East and North Africa	305 (8.7%)	–	–	–	–
South Asia	444 (12.6%)	–	–	–	–
Sub-Saharan Africa	156 (4.4%)	–	–	–	–
USA/Australia/New Zealand	61 (1.7%)	–	–	–	–
Unknown	8 (0.2%)	–	–	–	–
Years since landing					
Mean ± SD	17.30 ± 8.20	–	–	–	–
Median (IQR)	19 (11–24)	–	–	–	–
0–5 years	381 (10.8%)	–	–	–	–
5–9 years	320 (9.1%)	–	–	–	–
10+ years	2,812 (80.0%)	–	–	–	–

Screen-eligible population*	Immigrants (*n* = 1,604)	Long-term residents (*n* = 17,663)	Standardized difference	*p*-value	Total (*n* = 19,267)
FOBT screening (2 years)					
Eligible for FOBT (no scope in the past 10 years)	1,266 (89.9%)	14,055 (86.7%)	0.10	< .001	15,321 (87.0%)
No record of screening	775 (48.3%)	8,205 (46.5%)	0.04	0.288	8,980 (46.6%)
Up to date	396 (24.7%)	4,403 (24.9%)	0.01		4,799 (24.9%)
Ever screened	433 (27.0%)	5,055 (28.6%)	0.04		5,488 (28.5%)
Scope screening (10 years)					
No record of screening	1,301 (81.1%)	12,904 (73.1%)	0.19	< .001	14,205 (73.7%)
Up to date	279 (17.4%)	4,169 (23.6%)	0.15		4,448 (23.1%)
Ever Screened	24 (1.5%)	590 (3.3%)	0.12		614 (3.2%)
Any screening					
No record of screening	672 (41.9%)	6,392 (36.2%)	0.12	< .001	7,064 (36.7%)
Up to date	482 (30.0%)	5,813 (32.9%)	0.06		6,295 (32.7%)
Ever screened but overdue	450 (28.1%)	5,458 (30.9%)	0.06		5,908 (30.7%)

*ADG* adjusted diagnosis groups, *PCP* primary care provider, *FOBT* fecal occult blood test

*19,267 people in Ontario diagnosed with colorectal cancer between 2012 and 2017 who were eligible for screening and had at least 10 years of available data prior to diagnosis. Lookback period for screening was the date of diagnosis minus six months

**Fig. 2:**
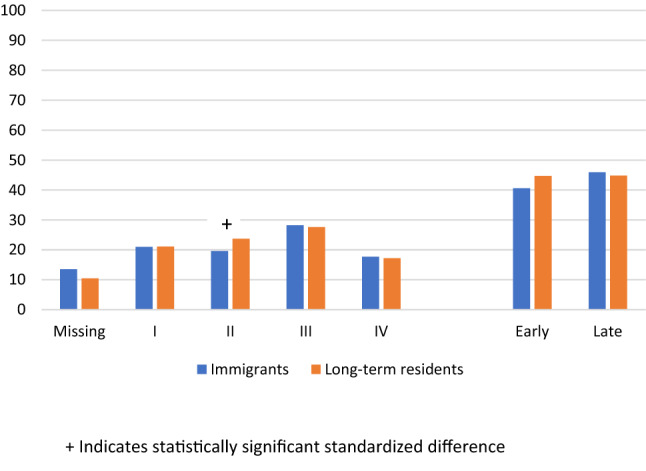
Percentage of immigrants (*n* = 3,513) and long-term residents (*n* = 34 204) at each stage of colorectal cancer diagnosis

For the overall cohort, 44.9% were diagnosed at a late stage, 44.3% at an early stage, and 10.8% were missing stage. Although immigrants were significantly less likely to be diagnosed at stage II and trended toward higher likelihood of missing stage (Fig. 2), overall no significant differences were seen between immigrants and long-term residents for stage at diagnosis (45.9% vs. 44.8% diagnosed at late stage, respectively, SD = 0.02). Age was associated with stage of diagnosis in bivariate analyses (Table 2), whereby people diagnosed at an early stage were more likely to be 50 years (the age of screening initiation) or older. Among both immigrants and long-term residents, those with early vs. late stage of diagnosis had more co-morbidities (immigrants: mean number of ADGs 6.81 vs. 6.11, SD = 0.22, long-term residents: mean 7.32 vs. 6.67, SD = 0.18). Having more primary care visits was associated with early stage of diagnosis for both groups and for immigrants only, those who were diagnosed at an early vs. late stage were more likely to have a high Usual Provider of Care index (55.6% vs. 49.8%, SD = 0.12). When limiting to immigrants, immigrant class, region of origin, and years since landing were not associated with stage of diagnosis. When limiting to the screen-eligible population (Table 2), people diagnosed at an early vs. late stage were less likely to have no record of screening (for immigrants: 39.6% vs. 45.3%, SD = 0.12, for long-term residents: 32.3% vs. 40.0%, SD = 0.16).

**Table 2:** Descriptive characteristics of study cohort by stage of diagnosis and immigrant status

Characteristics	Immigrants				Long-term residents			
	Early stage (*n* = 1,428)	Late stage (*n* = 1,612)	Standardized difference	*p* value	Early stage (*n* = 15,299)	Late stage (*n* = 15,320)	Standardized difference	*p* value
Sex								
Female	627 (43.9%)	735 (45.6%)	0.03	0.35	6,836 (44.7%)	6,913 (45.1%)	0.01	0.438
Male	801 (56.1%)	877 (54.4%)	0.03		8,463 (55.3%)	8,407 (54.9%)	0.01	
Age at diagnosis								
Mean ± SD	63.33 ± 13.89	61.70 ± 14.04	0.12	0.001	70.64 ± 12.36	68.66 ± 13.09	0.16	< .001
Median (IQR)	63 (53–74)	61 (51–72)	0.12	< .001	72 (63–80)	69 (60–79)	0.15	< .001
Age group								
< 50	214 (15.0%)	325 (20.2%)	0.14	0.002	774 (5.1%)	1,249 (8.2%)	0.12	< .001
50–74	877 (61.4%)	947 (58.7%)	0.05		8,178 (53.3%)	8,575 (56.0%)	0.05	
75–84	258 (18.1%)	262 (16.3%	0.05		4,404 (28.8%)	3,790 (24.7%)	0.09	
85 +	79 (5.5%)	78 (4.8%)	0.03		1,943 (12.7%)	1,706 (11.1%)	0.05	
Neighborhood income quintile*								
Quintile 1 (lowest)	382 (26.8%)	457 (28.3%)	0.04	0.443	2,987 (19.5%)	3,110 (20.3%)	0.02	0.297
Q2	296 (20.7%)	353 (21.9%)	0.03		3,209 (21.0%)	3,186 (20.8%)	0.00	
Q3	308 (21.6%)	307 (19.0%)	0.06		3,077 (20.1%)	3,109 (20.3%)	0.00	
Q4	262 (18.3%)	276 (17.1%)	0.03		3,000 (19.6%)	2,937 (19.2%)	0.01	
Q5 (highest)	177 (12.4%)	215 (13.3%)	0.03		3,003 (19.6%)	2,944 (19.2%)	0.01	
No. John’s Hopkins ADG co-morbidities								
Mean ± SD	6.81 ± 3.25	6.11 ± 3.20	0.22	< .001	7.32 ± 3.60	6.67 ± 3.56	0.18	< .001
Median (IQR)	7 (4–9)	6 (4–8)	0.21	< .001	7 (5–10)	6 (4–9)	0.18	< .001
0–5	541 (37.9%)	725 (45.0%)	0.14	< .001	5,122 (33.5%)	6,250 (40.8%)	0.15	< .001
6–9	292 (20.4%)	234 (14.5%)	0.16		4,079 (26.7%)	3,222 (21.0%)	0.13	
10 +	595 (41.7%)	653 (40.5%)	0.02		6,098 (39.9%)	5,848 (38.2%)	0.03	
No. PCP visits 6–30 months < index—all primary care providers								
Mean ± SD	8.48 ± 7.70	7.42 ± 7.66	0.14	< .001	7.86 ± 7.67	6.95 ± 7.32	0.12	< .001
Median (IQR)	7 (3–12)	6 (2–10)	0.17	< .001	6 (3–11)	5 (2–10)	0.16	< .001
No. PCP visits 6–30 months < index—patient’s usual provider of care								
Mean ± SD	6.23 ± 6.78	5.22 ± 5.93	0.16	< .001	5.99 ± 6.50	5.33 ± 6.35	0.10	< .001
Median (IQR)	5 (1–9)	4 (1–8)	0.15	< .001	4 (1–9)	3 (1–8)	0.14	< .001
UPC index								
Missing	29 (2.0%)	45 (2.8%)	0.05	0.005	432 (2.8%)	608 (4.0%)	0.06	< .001
0 visits	92 (6.4%)	130 (8.1%)	0.06		919 (6.0%)	1,134 (7.4%)	0.06	
1–2 visits	217 (15.2%)	301 (18.7%)	0.09		2,773 (18.1%)	3,056 (19.9%)	0.05	
UPC≤75%	296 (20.7%)	334 (20.7%)	0.00		2,528 (16.5%)	2,377 (15.5%)	0.03	
UPC > 75%	794 (55.6%)	802 (49.8%)	0.12		8,647 (56.5%)	8,145 (53.2%)	0.07	
PCP sex								
Missing	31 (2.2%)	52 (3.2%)	0.07	0.196	477 (3.1%)	671 (4.4%)	0.07	< .001
Female	478 (33.5%)	526 (32.6%)	0.02		4,361 (28.5%)	4,244 (27.7%)	0.02	
Male	919 (64.4%)	1,034 (64.1%)	0.00		10,461 (68.4%)	10,405 (67.9%)	0.01	
PCP country of training								
Missing	53 (3.7%)	85 (5.3%)	0.08	0.061	750 (4.9%)	919 (6.0%)	0.05	< .001
Canada	696 (48.7%)	740 (45.9%)	0.06		10,808 (70.6%)	10,498 (68.5%)	0.05	
Not Canada	679 (47.5%)	787 (48.8%)	0.03		3,741 (24.5%)	3,903 (25.5%)	0.02	
No. PCP visits 6–30 months < index—all primary care providers (spec = 00, 05)								
Mean ± SD	8.48 ± 7.70	7.42 ± 7.66	0.14	< .001	7.86 ± 7.67	6.95 ± 7.32	0.12	< .001
Median (IQR)	7 (3–12)	6 (2–10)	0.17	< .001	6 (3–11)	5 (2–10)	0.16	< .001
No. PCP visits 6–30 months < index—patient’s usual provider of care								
Mean ± SD	6.23 ± 6.78	5.22 ± 5.93	0.16	< .001	5.99 ± 6.50	5.33 ± 6.35	0.10	< .001
Immigrant category								
Economic immigrants	489 (34.2%)	571 (35.4%)	0.02	0.279				
Other immigrants	30 (2.1%)	44 (2.7%)	0.04					
Resettled refugee and protected person in Canada	228 (16.0%)	278 (17.2%)	0.03					
Sponsored family immigrants	681 (47.7%)	719 (44.6%)	0.06					
World bank region of origin								
East Asia and Pacific	444 (31.1%)	500 (31.0%)	0.00	0.444				
Europe and Central Asia	408 (28.6%)	505 (31.3%)	0.06					
Latin America and the Caribbean	177 (12.4%)	166 (10.3%)	0.07					
Middle East and North Africa	133 (9.3%)	144 (8.9%)	0.01					
South Asia	177 (12.4%)	194 (12.0%)	0.01					
Sub-Saharan Africa	57 (4.0%)	75 (4.7%)	0.03					
USA/Australia/New Zealand	28 (2.0%)	25 (1.6%)	0.03					
Unknown	≤5 (0.3%)	≤5 (0.2%)	0.02					
Years since landing								
Mean ± SD	17.42 ± 8.18	17.44 ± 8.03	0.00	0.935				
Median (IQR)	19 (11–24)	19 (11–24)	0.01	0.841				
0–5 years	149 (10.4%)	157 (9.7%)	0.02	0.808				
5–9 years	1,152 (80.7%)	1,313 (81.5%)	0.02					
10+ years	127 (8.9%)	142 (8.8%)	0.00					

Screen-eligible population	Immigrants				Long-term residents			
	Early stage	Late stage	Standardized difference	*p* value	Early stage	Late stage	Standardized difference	*p* value
Eligible for FOBT (no prior Scope)	606 (88.9%)	660 (90.9%)	0.07	0.201	6,742 (85.1%)	7,313 (88.2%)	0.09	< .001
FOBT screening (2 years)								
No record of screening	313 (45.9%)	372 (51.2%)	0.11	0.006	3,414 (43.1%)	4,085 (49.3%)	0.12	< .001
Up to date	162 (23.8%)	188 (25.9%)	0.05		2,110 (26.6%)	1,948 (23.5%)	0.07	
Ever Screened but overdue	207 (30.4%)	166 (22.9%)	0.17		2,398 (30.3%)	2,257 (27.2%)	0.07	
Scope screening (10 years)								
No record of screening	535 (78.4%)	615 (84.7%)	0.16	0.008	5,543 (70.0%)	6,394 (77.1%)	0.16	< .001
Up to date	137 (20.1%)	101 (13.9%)	0.16		2,078 (26.2%)	1,649 (19.9%)	0.15	
Ever Screened but overdue	10 (1.5%)	10 (1.4%)	0.01		301 (3.8%)	247 (3.0%)	0.05	
Any CRC screening								
No record of screening	270 (39.6%)	329 (45.3%)	0.12	0.008	2,562 (32.3%)	3,313 (40.0%)	0.16	< .001
Up to date	199 (29.2%)	223 (30.7%)	0.03		2,763 (34.9%)	2,542 (30.7%)	0.09	
Ever Screened but overdue	213 (31.2%)	174 (24.0%)	0.16		2,597 (32.8%)	2,435 (29.4%)	0.07	

*ADG* adjusted diagnosis groups, *PCP* primary care provider, *FOBT* fecal occult blood test

*Neighborhood income quintile was missing for a total of 7 immigrants and 71 long-term residents. Not included in table due to small cell sizes

**Fig. 3:**
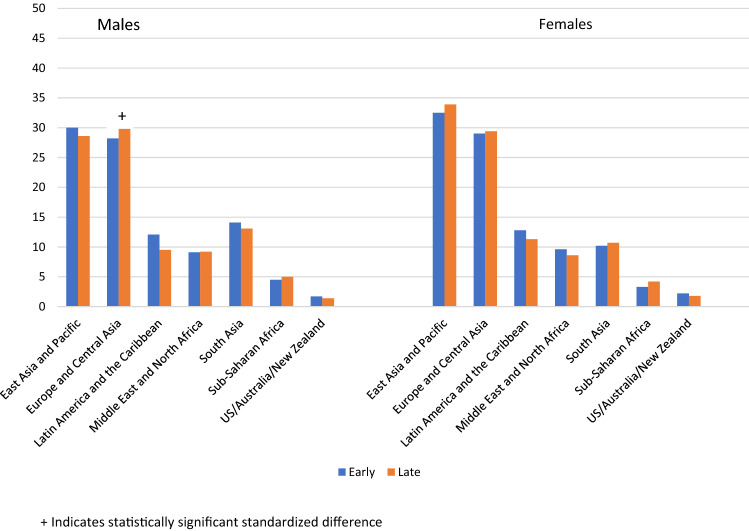
Percentage of immigrant males (*n* = 1,678) and immigrant females (*n* = 1,362) with early and late stage of diagnosis by sex and region of origin. Among males, 801 were diagnosed early stage vs. 877 diagnosed late stage. Among women, 627 were diagnosed early stage vs. 735 diagnosed late stage. +Indicates statistically significant standardized difference

Among the 3040 immigrants with a known stage of diagnosis (1678 men and 1362 women), there were generally no differences seen when examining early versus late stage of diagnosis among immigrants by both sex and region of origin (Fig. 3). A notable exception was seen for men from Europe and Central Asia; 28.2% (226/801) of immigrant men diagnosed at an early stage were from this region vs. 33.0% (289/877) of men diagnosed at a late stage (SD = 0.10). Men from Latin America and the Caribbean trended toward a higher likelihood of early-stage diagnosis; 12.1% (97/801) of immigrant men diagnosed at an early stage were from this region versus 9.5% (83/877) of men diagnosed at a late stage (SD = 0.09).

In our multivariable analyses (Table 3), immigrant status was statistically significantly associated with a late-stage diagnosis in the unadjusted model (relative risk of late-stage diagnosis 1.06 [95% CI 1.02–1.10]). However, immigrant status was no longer significant after adjusting for age and sex nor in our fully adjusted model (ARR 1.01 [95% CI 0.98–1.05]. In the fully adjusted model, increasing ADG category was associated with lower likelihood of late-stage diagnosis (ARR 0.86 [95% CI 0.83–0.89] for 10 or more ADGs versus 0–5 ADGs) and having no visits to primary care (versus a high level of continuity of care) was associated with a higher likelihood of late-stage diagnosis (ARR 1.07 [95% CI 1.03–1.12]). When the model was repeated limiting to the screen-eligible population and including screening status in the model, results were similar (data not shown); immigrant status remained non-significant (ARR 0.99 [95% CI 0.91–1.07]). As compared to those with no record of screening in available data, those who were up to date on screening had an ARR of late-stage diagnosis of 0.90 [95% CI 0.86–0.95] and those who had been screened but were overdue had an ARR of 0.89 [95% CI 0.85–0.94] in the fully adjusted model.

**Table 3:** Results from multivariable model using Poisson regression. Adjusted relative risks represent late vs. early stage of diagnosis

Variables	Relative risk [95% confidence interval]
*Unadjusted*	
Immigrant (vs. long-term resident)	1.06 [1.02–1.10]
*Age and sex-adjusted*	
Immigrant (vs. long-term resident)	1.02 [0.98–1.05]
Male (vs. female)	0.98 [0.96–1.00]
Age (as continuous variable)	0.99 [0.99–1.00]
*Full model*	
Immigrant (vs. long-term resident)	1.01 [0.98–1.05]
Male (vs. female)	0.97 [0.94–0.99]
Age (as continuous variable)	1.00 [1.00–1.00]
Neighborhood income quintile (quintile 5 as reference group)	
Income quintile 1 (lowest)	1.03 [1.00–1.07]
Income quintile 2	1.01 [0.98–1.05]
Income quintile 3	1.01 [0.98–1.04]
Income quintile 4	1.00 [0.96–1.03]
Co-morbidities (0–5 ADGs as reference group)	
6–9 ADGs	0.93 [0.91–0.95]
10+ ADGs	0.86 [0.83–0.89]
Primary care visits in the 6–30 months prior to diagnosis (as continuous variable)	1.00 [1.00–1.00]
Continuity of care (Usual provider of care index of 75% or greater as reference group)	
0 visits to primary care	1.07 [1.03–1.12]
1–2 visits to primary care	1.01 [0.98–1.04]
Usual provider of care index less than 75%	1.01 [0.98–1.04]
Male (vs female) primary care physician	1.02 [1.00–1.05]
Missing sex (vs female) primary care physician	1.10 [0.99–1.22]
Non-Canadian (vs. Canadian) medical school for primary care physician	1.04 [1.02–1.07]
Missing country (vs. Canadian) for medical school for primary care physician	0.97 [0.89–1.05]

## Discussion

In this population-based retrospective cohort study of more than 37,000 people in Ontario diagnosed with CRC from 2012 to 2017, we found that almost 45% were diagnosed with their cancer at a late stage, and we identified 9.7% of our cohort as immigrants. Immigrants with CRC were more likely to have a late-stage diagnosis than their long-term resident counterparts (ARR 1.06 [95% CI 1.02–1.10]), but after adjusting for age, sex, and other sociodemographic and healthcare-related variables, this difference was no longer significant (ARR 1.01 [95% CI 0.98–1.05]). Factors related to primary care contact and continuity of care played a role in stage of diagnosis, whereby people with no primary care visits versus those with high primary care continuity were more likely to have a late-stage diagnosis. Number of co-morbidities was inversely associated with late-stage diagnosis in the fully adjusted model, with an ARR of 0.86 [95% CI 0.83–0.89] for those with 10 or more ADGs versus those with 0–5 ADGs.

Despite a lower likelihood of CRC screening among immigrants and despite screening providing a diagnostic stage advantage, we found that immigrants in Ontario were not more likely to be diagnosed with CRC at a late stage after adjusting for age, sex, and healthcare-related variables. The reasons for this finding cannot be ascertained from this study, but it is possible that the ‘healthy immigrant effect’ plays a role. The healthy immigrant effect refers to immigrants being in better physical condition on arrival than host country inhabitants due to selective migration processes. In Canada, most immigrants must submit to a medical examination to ensure they do not burden Canada’s health and social services system [28]. Canadian studies have found that morbidity and mortality due to chronic diseases are lower among immigrants than among the general population and in Ontario, immigrants have lower incidence of CRC [11, 29, 30]. Differences in risk factors for CRC between immigrants and long-term residents may also play a role in our findings, and these risk factors may themselves also be related to the healthy immigrant effect. Risk factors for CRC include high body mass index, red meat intake, cigarette smoking, low physical activity, and low fruit and vegetable consumption, and it is possible that immigrants in Ontario are less likely to exhibit these risk factors [30–33]. Of note, CRC incidence rates are approximately threefold higher in high-income countries than lower-income countries, with the highest rates of CRC globally in parts of Europe [34]. Incidence of CRC tends to rise in countries as they become more developed, further pointing to the potential influence of lifestyle factors. In line with these international findings, we found men from European and Central Asian countries to have higher likelihood of late-stage diagnosis. Immigrants from this region have also been found to have the lowest rates of CRC screening in Ontario among immigrant groups and to be the only immigrant group with an adjusted risk of developing CRC greater than that of Canadian-born [10, 11].

Our findings confirm the important role that primary care plays in early diagnosis of CRC. We found that cancer screening led to an earlier stage of diagnosis, and screening in Ontario is typically recommended and coordinated by primary care physicians. Primary care physicians also coordinate investigations and referrals to specialists to follow-up on symptoms. A relationship with a primary care physician may be especially important for immigrants, for whom having a high Usual Provider of Care index (i.e., high continuity of care) was associated with a higher likelihood of early-stage CRC diagnosis in bivariate analyses. Primary care physicians are expected to advocate for their patients and help them to navigate the healthcare system [35], and this advocacy role may be even more important for non-Canadian-born patients than others as they deal with the social determinants of health, navigating a potentially unfamiliar healthcare system and systemic discrimination [36–39]. Interestingly, in their Ontario study of women with breast cancer, Walsh et al. similarly found that higher continuity of primary care was associated with a shorter time from primary care presentation to initiation of chemotherapy, but only for immigrant women [40].

In both bivariate and multivariable analyses, we found that those with the fewest co-morbidities had the highest likelihood of late-stage diagnosis. Other Ontario studies have found that those with the lowest number of co-morbidities had lower rates of cancer screening and lower rates of screening for hyperlipidemia [41–43]. Taken together, these findings suggest that people with few co-morbidities may have less contact with the healthcare system in general, which paradoxically may negatively affect their chances of regularly receiving preventive care including cancer screening and negatively affecting their chances of warning signs of CRC being detected early by healthcare providers.

We found that immigrants were diagnosed with CRC at younger ages than long-term residents in Ontario. Although evidence from both Canada and the USA have indicated a shift toward younger age of diagnosis for CRC [44, 45], findings from the USA indicate that this trend is driven by non-Hispanic Whites [45] and it is possible that our findings are at least partially explained by the fact that the long-term residents group included immigrants who arrived before 1985 and are thus more likely to be older.

This study is strengthened by its population-level approach and use of multiple linked administrative databases with rich data. However, it also has several limitations. First, the IRCC-PR database does not include immigrants who arrived in Ontario before 1985 or who first came to Ontario from another province or territory. These two groups of immigrants would have been misclassified in the long-term resident group, and as noted, the former may partially explain why immigrants in our cohort were noticeably younger than long-term residents. Second, the most recent immigrants, those in Ontario for less than three years, and immigrants without documentation and thus not in the IRCC-PR database were not included in the study. However, these numbers are likely to be relatively small. Third, CRC screening in Ontario has steadily increased over time with the establishment of the organized provincial screening program in 2008 [6, 46]. Ontario also transitioned from the use of FOBT to FIT in 2019, which is easier for patients to complete [47]. Uptake of CRC screening in our study results may not reflect current rates, suggesting that this study should be repeated in the future after FIT is well established. Fourth, we are not able to determine from study data who in our cohort was at elevated risk of CRC due to family history or relevant hereditary syndromes and thus may receive screening at levels not recommended for the average-risk population. In the USA and Canada, it has been estimated that 20–30% of patients with CRC have a family history and 2–5% have a hereditary syndrome [48–50]. Fifth, we did not examine the interaction between continuity of care and co-morbidity status with provider characteristics (e.g., sex and country of training). This relationship should be explored in future research. Finally, we were not able to examine the role of race/ethnicity in this study as these data are not readily available or systematically collected in Ontario.

## Conclusion

In the context of an organized provincial screening program, we found that 44.9% of people in Ontario diagnosed with CRC from 2012 to 2017 were diagnosed at a late stage. Immigrants were slightly more likely to be diagnosed at a late stage, but this difference was no longer significant after adjusting for age, sex, and healthcare-related characteristics. However, men from Europe and Central Asia were significantly more likely to be diagnosed at a late stage, which warrants further investigation and intervention, as does the potentially earlier age at diagnosis for immigrants. For all Ontarians, but for Ontarians born outside of Canada in particular, attachment to a primary care provider who provides regular preventive care should be a healthcare system priority in order to help further shift the population to earlier stage of diagnosis.

## Data Availability

The dataset from this study is held securely in coded form at ICES. While legal data sharing agreements between ICES and data providers (e.g., healthcare organizations and government) prohibit ICES from making the dataset publicly available, access may be granted to those who meet pre-specified criteria for confidential access, available at www.ices.on.ca/DAS (email: das@ices.on.ca). The full dataset creation plan and underlying analytic code are available from the authors upon request, understanding that the computer programs may rely upon coding templates or macros that are unique to ICES and are therefore either inaccessible or may require modification.
